# Endothelial-Mesenchymal Transition of Brain Endothelial Cells: Possible Role during Metastatic Extravasation

**DOI:** 10.1371/journal.pone.0119655

**Published:** 2015-03-05

**Authors:** István A. Krizbai, Ákos Gasparics, Péter Nagyőszi, Csilla Fazakas, Judit Molnár, Imola Wilhelm, Rita Bencs, László Rosivall, Attila Sebe

**Affiliations:** 1 Institute of Biophysics, Biological Research Centre, Hungarian Academy of Sciences, Temesvári krt. 62, 6726, Szeged, Hungary; 2 Institute of Life Sciences, Vasile Goldis Western University of Arad, Liviu Rebreanu Str. 86, 310414, Arad, Romania; 3 Department of Pathophysiology, Semmelweis University, Nagyvárad Square 4, 1089, Budapest, Hungary; 4 Pediatrics and Nephrology Research Group, Hungarian Academy of Sciences and Semmelweis University, Nagyvárad Square 4, 1089, Budapest, Hungary; Thomas Jefferson University, UNITED STATES

## Abstract

Cancer progression towards metastasis follows a defined sequence of events described as the metastatic cascade. For extravasation and transendothelial migration metastatic cells interact first with endothelial cells. Yet the role of endothelial cells during the process of metastasis formation and extravasation is still unclear, and the interaction between metastatic and endothelial cells during transendothelial migration is poorly understood. Since tumor cells are well known to express TGF-β, and the compact endothelial layer undergoes a series of changes during metastatic extravasation (cell contact disruption, cytoskeletal reorganization, enhanced contractility), we hypothesized that an EndMT may be necessary for metastatic extravasation. We demonstrate that primary cultured rat brain endothelial cells (BEC) undergo EndMT upon TGF-β1 treatment, characterized by the loss of tight and adherens junction proteins, expression of fibronectin, β1-integrin, calponin and α-smooth muscle actin (SMA). B16/F10 cell line conditioned and activated medium (ACM) had similar effects: claudin-5 down-regulation, fibronectin and SMA expression. Inhibition of TGF-β signaling during B16/F10 ACM stimulation using SB-431542 maintained claudin-5 levels and mitigated fibronectin and SMA expression. B16/F10 ACM stimulation of BECs led to phosphorylation of Smad2 and Smad3. SB-431542 prevented SMA up-regulation upon stimulation of BECs with A2058, MCF-7 and MDA-MB231 ACM as well. Moreover, B16/F10 ACM caused a reduction in transendothelial electrical resistance, enhanced the number of melanoma cells adhering to and transmigrating through the endothelial layer, in a TGF-β-dependent manner. These effects were not confined to BECs: HUVECs showed TGF-β-dependent SMA expression when stimulated with breast cancer cell line ACM. Our results indicate that an EndMT may be necessary for metastatic transendothelial migration, and this transition may be one of the potential mechanisms occurring during the complex phenomenon known as metastatic extravasation.

## Introduction

Endothelial-mesenchymal transition (EndMT) is an embryonic program necessary for organ development. Despite being normally dormant in adult organisms, this mechanism can be reactivated during several pathological conditions, such as cancer and fibrosis. At cellular and molecular level EndMT is regulated by similar factors and signaling pathways under both physiological and pathological conditions. EndMT was first described during heart development [1]. During cancer, EndMT contributes to the formation of cancer-associated fibroblasts [2], and it was found to be an important mechanism during renal and cardiac fibrosis [3, 4]. Recently, EndMT was found to be involved in the formation of cerebral cavernous malformations in CCM1 deficient mice [5].

EndMT is related to epithelial-mesenchymal transition, which represents a highly similar mechanism characterized by analogous sequence of events. During EndMT endothelial cells lose their endothelial markers and endothelial cell contacts (e.g., VE-cadherin), express fibroblast-specific and mesenchymal proteins (e.g., FSP1, PAI-1), start to synthesize extracellular matrix (e.g., fibronectin), and ultimately differentiate into α-smooth muscle actin (SMA)-positive myofibroblasts. EndMT follows a sequentially orchestrated, defined chronology: down-regulation of the endothelial program, activation of the mesenchymal-fibrogenic program, and finally the activation of the myogenic program [6, 7].

Metastasis formation is responsible for the overwhelming majority of cancer-related mortality [8]. Cancer progression towards metastasis follows a defined sequence of events described as the metastatic cascade. First, cells from the primary tumors invade the local extracellular matrix, then intravasate into the lumina of blood vessels. Following the transport through the vasculature metastatic cells extravasate into the surrounding tissue, form micrometastasis in the target tissue and, by reinitiating their proliferative program, generate macroscopic metastases [9, 10]. Despite the fact that the metastatic cascade is a highly inefficient process, large numbers of circulating tumor cells can undergo extravasation [11].

In order to overcome physical barriers extravasating tumor cells secrete factors that reduce endothelial barrier function. Tumor cells are also well known to express TGF-β1 [12, 13], whereas malignant melanoma patients present elevated plasma TGF-β1 and TGF-β2 levels [14], breast cancer cell lines also expressing different TGF-β isoforms [15]. In the context of metastatic progression, serum TGF-β1 levels showed a sudden elevation at the time point of metastasis initiation [16]. Extravasation takes place mainly through paracellular transendothelial migration (TEM). Cancer cells activate signaling pathways in endothelial cells via secreted factors to disrupt VE-cadherin complexes. This enables the interendothelial junctional complex disintegration, and metastatic cells migrate through the endothelial cell junction openings [17, 18].

The majority of intracranial tumors are brain metastases, primary brain tumors representing only about 10% of new cases of intracranial malignancies [19]. Brain metastases mainly originate from lung cancer, breast cancer and malignant melanoma [20]. Since the central nervous system (CNS) lacks a lymphatic system, metastatic cells can only reach the brain through the blood stream. In order to invade the CNS parenchyma, cancer cells need to pass the blood-brain barrier (BBB), which represents the tightest endothelial barrier in the organism. In spite of this, the role of endothelial cells during the process of metastasis formation and extravasation is still unclear, and the interaction between metastatic and endothelial cells during transendothelial migration is poorly understood [21]. During metastasis endothelial cells undergo expressional changes and signaling events corresponding to a transition towards a mesenchymal phenotype: rearrangements in cell surface and cell contact proteins or enhanced contractility. Therefore, we hypothesized that EndMT is necessary for extravasation of metastatic cells. Here we demonstrate that primary brain endothelial cells (BECs) undergo EndMT upon TGF-β1 treatment in vitro, and activated cancer cell line conditioned medium is sufficient to induce EndMT of BECs in a TGF-β-dependent manner. Moreover, stimulation of endothelial cells with activated cancer cell line conditioned medium resulted in TGF-β-dependent decrease of transendothelial electrical resistance, increase in adhesion between metastatic and endothelial cells and enhanced transendothelial migration of melanoma cells. These effects were not confined to brain endothelial cells; human umbilical vein endothelial cells (HUVEC) could also undergo EndMT under similar conditions. Our results indicate that an EndMT may be necessary for metastatic transendothelial migration, and this transition may be one of the potential mechanisms occurring during the complex phenomenon known as metastatic extravasation.

## Materials and Methods

### Cell culture and treatments

Primary rat brain endothelial cells (RBECs) were isolated from two week old Wistar rats (Toxi-Coop, Budapest, Hungary), as described previously [22]. Cerebral cortices were disaggregated and digested in two steps with collagenase (Sigma, Budapest, Hungary) and collagenase/dispase (Roche, Budapest, Hungary) followed by centrifugation for 10 min at 1000xg on Percoll (Sigma) gradient; microvessel fragments were collected and plated onto fibronectin/collagen-coated dishes. Endothelial cell outgrowth was cultured in DMEM/F12 (Life Technologies, Budapest, Hungary) containing 10% plasma-derived serum (PDS, First Link, Wolverhampton, UK), bFGF, heparin, and insulin-transferrin-selenite (Sigma). Isolation of primary cerebral endothelial cells was carried out in strict accordance with the national and international recommendations for the care and use of laboratory animals. The protocol was reviewed and approved by the Regional Animal Health and Food Control Station of Csongrád County (Permit Number: XVI/2980/2012).

Human umbilical vein endothelial cells (HUVECs) were isolated as described previously [23]. Human umbilical cord veins were obtained from the 2^nd^ Department of Obstetrics and Gynaecology, Semmelweis University, after obtaining written informed consent for use of these samples in research approved by the Semmelweis University Regional and Institutional Committee of Science and Research Ethics, Budapest, Hungary (TUKEB 126/2014). Briefly, HUVECs were separated by collagenase treatment (Sigma). Cells were seeded onto 0.5% gelatin-coated flasks (Sigma) and cultured in M199 medium (Sigma) supplemented with 15% fetal bovine serum (FBS, Life Technologies), 100 IU/ml penicillin (Life Technologies), 100 μg/ml streptomycin (Life Technologies), 7.5 IU/ml heparin (Merckle, Ulm, Germany), 2 ng/ml epidermal growth factor (R&D Systems, Abington, UK), and 250 pg/ml β-endothelial cell growth factor (R&D Systems), referred to as complete medium. Cells from passages 2–4 were used for experiments.

B16/F10 murine melanoma cells were kept in RPMI medium (Sigma) supplemented with 5% FBS (Lonza, Basel, Switzerland) and Glutamax (Life Technologies). A2058 human melanoma cells were maintained in MEM (Sigma) supplemented with 5% FBS (Lonza) and Glutamax (Life Technologies). SK-BR3, MCF-7 and MDA-MB231 human breast cancer cells were grown in DMEM (Sigma) supplemented with 10% FBS (Lonza). Cells were grown at 37°C under a humidified atmosphere containing 5% CO_2_.

TGF-β1 (Sigma) treatments were carried out as specified at the individual experiments (10 ng/ml or vehicle for controls).

To obtain cancer cell line conditioned medium for RBECs, serum-free DMEM/F12 medium was collected after 24 hrs. Latent TGF-β was heat activated (80°C, 10 min). PDS was supplemented before adding non activated or activated conditioned medium to RBECs. To obtain cancer cell line conditioned medium for HUVECs, serum-free M199 medium was collected after 24 hrs. Latent TGF-β was heat activated (80°C, 10 min). FBS was supplemented before adding non activated or activated conditioned medium to HUVECs.

### Antibodies and reagents

The following commercially available antibodies were used: claudin-5 (Zymed/Life Technologies), occludin (Transduction Laboratories, Franklin Lakes, NJ, USA), VE-cadherin (Cell Signaling, Danvers, MA, USA), N-cadherin (Transduction Laboratories), β1-integrin (Santa Cruz Biotechnologies, CA, USA), fibronectin (Sigma), calponin (DAKO, Glostrup, Denmark), SMA (Sigma), α-tubulin (Sigma), SRF (Santa Cruz Biotechnologies), phospho-Smad2/Smad3 (Cell Signaling), Smad2/3 (Cell Signaling). Peroxidase-conjugated anti-mouse and anti-rabbit secondary antibodies were obtained from Cell Signaling. Cy3-labeled anti-mouse and Cy3-labeled anti-rabbit antibodies were obtained from Jackson ImmunoResearch (Newmarket, UK). SB-431542 was purchased from Sigma. Y-27632 was obtained from Tocris (Bristol, UK). For inhibitor studies control cells were treated with vehicle.

### Immunofluorescence microscopy

Cells were fixed with ethanol/acetic acid (95/5) at −20°C for 5 min. After blocking with 3% BSA for 30 min, samples were incubated with primary antibodies. The staining was visualized using Cy3-conjugated secondary antibodies. Nuclear staining of the cells was carried out using Hoechst 33342 (Sigma). Images were recorded by a Nikon Eclipse TE2000U photomicroscope with epifluorescent capabilities connected to a digital camera (Spot RT KE).

### Western Blot

Cells were scraped into RIPA lysis buffer. Protein concentration was determined using the BCA Protein Assay (Pierce Thermo Scientific, Rockford, IL). Samples were mixed in a 1:1 ratio with two times Laemmli buffer and boiled for 5 minutes. Equal amounts of protein were separated on 12% SDS-polyacrylamide gel and transferred to nitrocellulose membranes (Bio-Rad, Budapest, Hungary). Membranes were blocked with Tris-buffered saline, containing 0.1% Tween 20 and 5% skim milk for an hour, and then incubated overnight with the primary antibody (in Tris-buffered saline-Tween 20 plus 0.5% skim milk), extensively washed, and incubated with the corresponding peroxidase-conjugated secondary antibody. Blots were visualized by the electrochemiluminescence detection system (Thermo Scientific, Waltham, MA, USA). Quantification results for N-cadherin are presented as mean ± SE.

### Transendothelial Electrical Resistance Measurement (TEER)

RBECs were grown on collagen/fibronectin-coated semipermeable filters (0.4 μm pore size, 1.12 cm^2^, Costar Corning Transwell Clear, Sigma). After reaching confluence, the endothelial monolayer was supplied with 550 nM hydrocortisone, 250 μM CPT-cAMP (Sigma) and 17.5 μM RO-201724 (Roche) and placed into the wells of the CellZscope instrument (nanoAnalytics, Münster, Germany) containing astrocyte conditioned medium. Treatments were applied after TEER had reached plateau. TEER was recorded at the indicated time points.

### Adhesion experiments

RBECs were grown in 24-well plates. After reaching confluence RBECs were treated with TGF-β1, B16/F10 conditioned media (B16/F10-CM) or B16/F10 activated conditioned media (B16/F10-ACM) in the presence or absence of SB-431542 for 5 hrs. B16/F10 melanoma cells were fluorescently labeled using Oregon Green 488 carboxylic acid diacetate succinimidyl ester (Life Technologies) using the protocol supplied by the manufacturer. 5x10^4^ melanoma cells/well were loaded onto the endothelial monolayer in serum-free medium and left for 70 min. After washing cells were fixed using ethanol/acetic acid (95/5) at-20°C for 5 min. Melanoma cells attached to endothelial cells were photographed and counted using the Image-Pro Plus software (Media Cybernetics, Rockville, MD, USA).

### Transmigration of melanoma cells through BEC monolayers

RBECs were cultured until confluence in 12 well plates and treated with TGF-β1 or ACM for 5 h. B16/F10 melanoma cells (2 x 10^4^/well) were plated onto the monolayer. For inhibitor studies, cells were preincubated with SB-431542 for 60 minutes. Cells were monitored over 10 h and phase contrast images were taken using an Andor NEO sCMOS camera (Andor Technology, Belfast, UK) connected to the Nikon Eclipse Ti-E inverted microscope equipped with a home built incubator set to 37°C. Photographs were made every 5 min and transmigrated cells were counted.

### Gene microarray data analysis

For this a set of gene expression profiles was downloaded from Gene Expression Omnibus (GEO) of the National Center for Biotechnology Information (NCBI). Data was derived from HUVECs and 1205Lu melanoma cells grown alone or in co-culture (accession number: GSE8699). Gene expression levels between HUVECs grown alone and HUVECs co-cultured with the melanoma cells were analyzed. EndMT marker gene expression levels were compared in the two datasets. Genes with a detection p-value <0.05 were considered if presenting a minimum fold change threshold of 1.5.

## Results

### 1. TGF-β1 induces EndMT of cerebral endothelial cells

**Fig 1:**
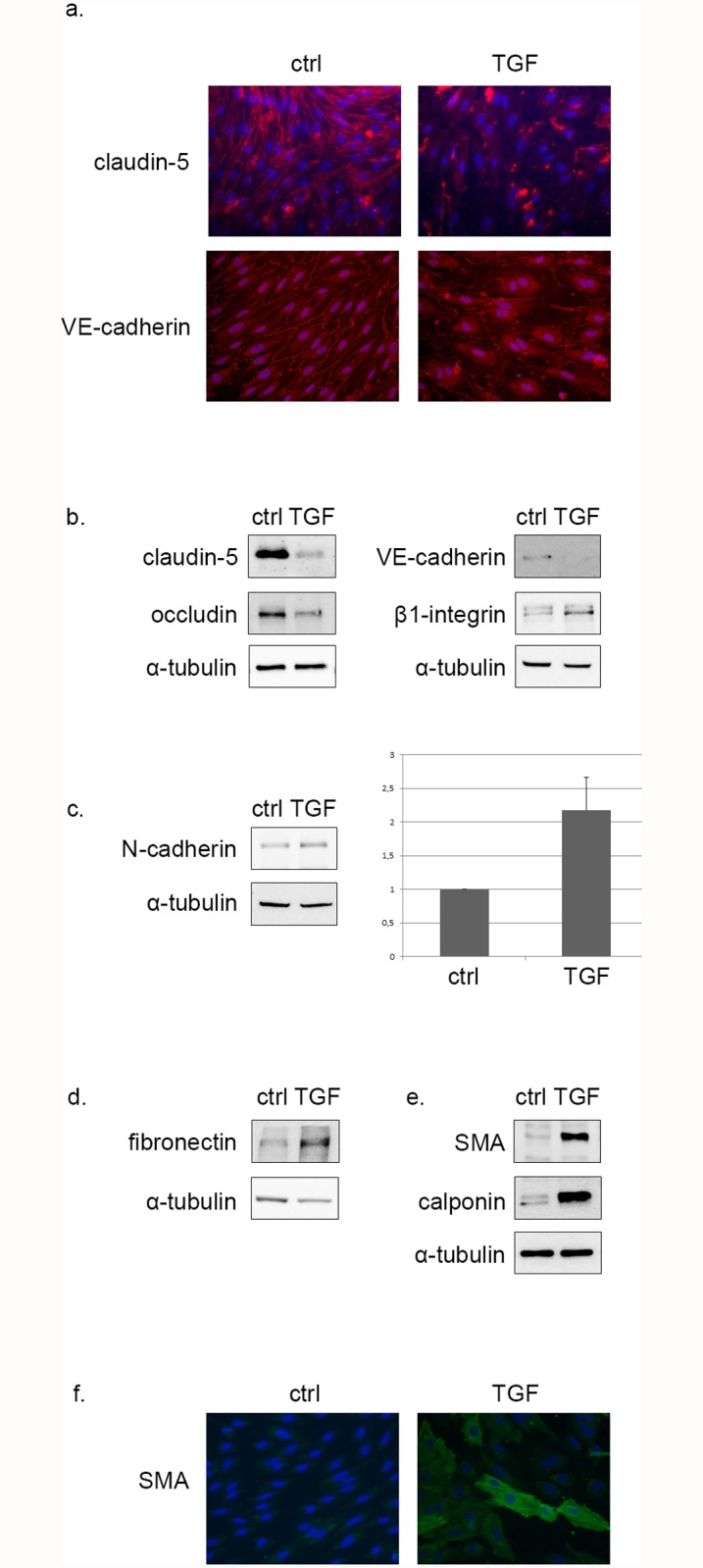
TGF-β1 induces EndMT in primary rat BECs. **(a)** BECs were subjected to 48 hrs TGF-β1 treatment, then fixed and stained for claudin-5 and VE-cadherin. Control cells showed claudin-5 and VE-cadherin at their intercellular borders, which was depleted in TGF-β1 treated cells (400x magnification). BECs were treated with TGF-β1 for 48 hrs, and were analyzed for endothelial, mesenchymal and myogenic marker expression by Western blot. TGF-β1 treatment led to the **(b)** down-regulation of tight and adherens junction protein expression (claudin-5, occludin, VE-cadherin), it induced β1-integrin expression and **(c)** and led to a marked increase in N-cadherin expression (2.18±0.48 fold based on three independent experiments, where N-cadherin expression increased 2.11, 1.52 and 2.89 fold respectively, as compared to controls). TGF-β1 treated cells showed a robust expression of fibronectin **(d)**, SMA and calponin **(e)**. SMA expression was observed by immunofluorescence microscopy (400x magnification) under similar conditions as well **(f)**.

To assess whether primary rat BECs (RBEC) could undergo transition towards a mesenchymal phenotype, RBECs were treated with 10 ng/ml of TGF-β1, and the subsequent changes were examined by immunofluorescence microscopy and Western blot. First, to visualize potential loss of cell-cell contacts, cells were immunostained for claudin-5 and VE-cadherin. In control cells claudin-5 and VE-cadherin accumulated in the cell membrane, clearly delimitating neighboring cells. This peripheral staining disappeared 48 hrs after TGF-β1 treatment and RBECs became negative for claudin-5 and VE-cadherin expression (Fig. 1a).

As loss of cell contacts is a common feature of endothelial-mesenchymal transition, the effects of TGF-β1 treatment with regard to specific EndMT markers was examined. Similarly to EMT, EndMT markers can be classified according to the different programs activated during transition: down-regulation of the endothelial program characterized by the loss of tight and adherent junctions, activation of the mesenchymal-fibroblast program as evidenced by mesenchymal marker expression, and the activation of the myogenic program ensuring enhanced contractility.

TGF-β1 treatment induced a severe decline in tight and adherent junction protein expression. TGF-β1 induced down-regulation of claudin-5, occludin-1 and VE-cadherin expression. Concomitantly TGF-β1 induced β1-integrin expression (Fig. 1b). Endothelial cells are known to express N-cadherin, yet upon TGF-β1 treatment RBECs exhibited a significant increase in N-cadherin expression (Fig. 1c). Following TGF-β1 treatment RBECs started expressing fibronectin (Fig. 1d). Finally, we observed de novo expression of calponin and α-smooth muscle actin (SMA), myogenic expression patterns ensuring enhanced endothelial cell contractility (Fig. 1e, 1f). SMA expression, an established myofibroblast marker, reflects the last stage of EndMT. Based on these data we concluded that TGF-β1 treatment induces EndMT in RBECs.

**Fig 2:**
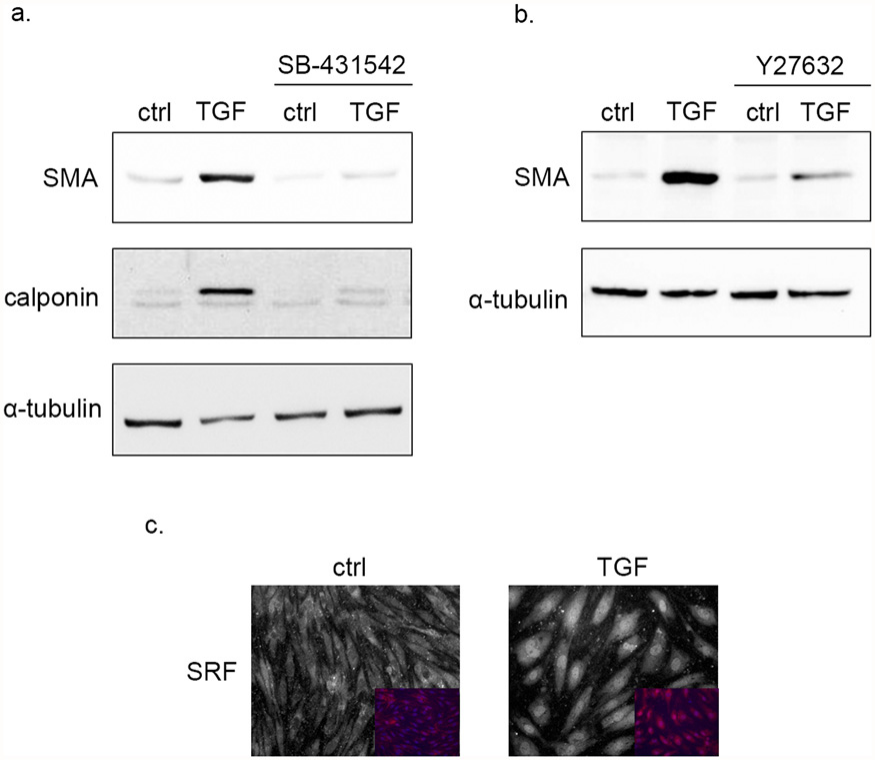
TGF-β1 induced SMA expression in BECs requires TGFβR and ROCK. TGF-β1 treatment leads to SRF nuclear translocation. **(a)** Cells were preincubated with 10 μM SB-431542 for 60 min and then treated with TGF-β1 for 48 hrs in the presence of SB-431542, and analyzed by Western blot. TGF-β1 induced SMA and calponin expression was mitigated in SB-431542 treated cells. **(b)** Cells were pretreated with 10 μM Y-27632 for 60 min and then treated with TGF-β1 for 48 hrs in the presence of the inhibitor, and analyzed by Western blot. TGF-β1 induced SMA expression was inhibited in the presence of Y-27632. **(c)** BECs were subjected to 24 hrs of TGF-β1 treatment, then were fixed and stained for SRF. Specimens were analyzed by immunofluorescence microscopy. Control cells expressed SRF mainly in their cytoplasm, whereas TGF-β1 treatment led to its nuclear translocation (400x magnification).

To examine the specificity of TGF-β1 treatment we used SB-431542, a well-known TGF-β receptor I kinase inhibitor. Treatment of cells with the inhibitor prevented TGF-β1 induced SMA and calponin expression (Fig. 2a). The Rho-SRF (Serum Response Factor) pathway is a central regulator of SMA expression during EMT. The following experiments were carried out to determine whether this pathway could be relevant for SMA expression during EndMT of RBECs. Cells were pretreated with the specific Rho kinase inhibitor Y-27632. Y-27632 prevented TGF-β1 induced SMA expression (Fig. 2b). Moreover, TGF-β1 induced a marked nuclear translocation of SRF in RBECs (Fig. 2c), a translocation necessary for CArG-box dependent protein expression. These results indicated that Rho kinase is required for mediating TGF-β1-induced effects.

### 2. Cancer cell line conditioned/activated medium induces TGF-β-dependent EndMT in brain endothelial cells

**Fig 3:**
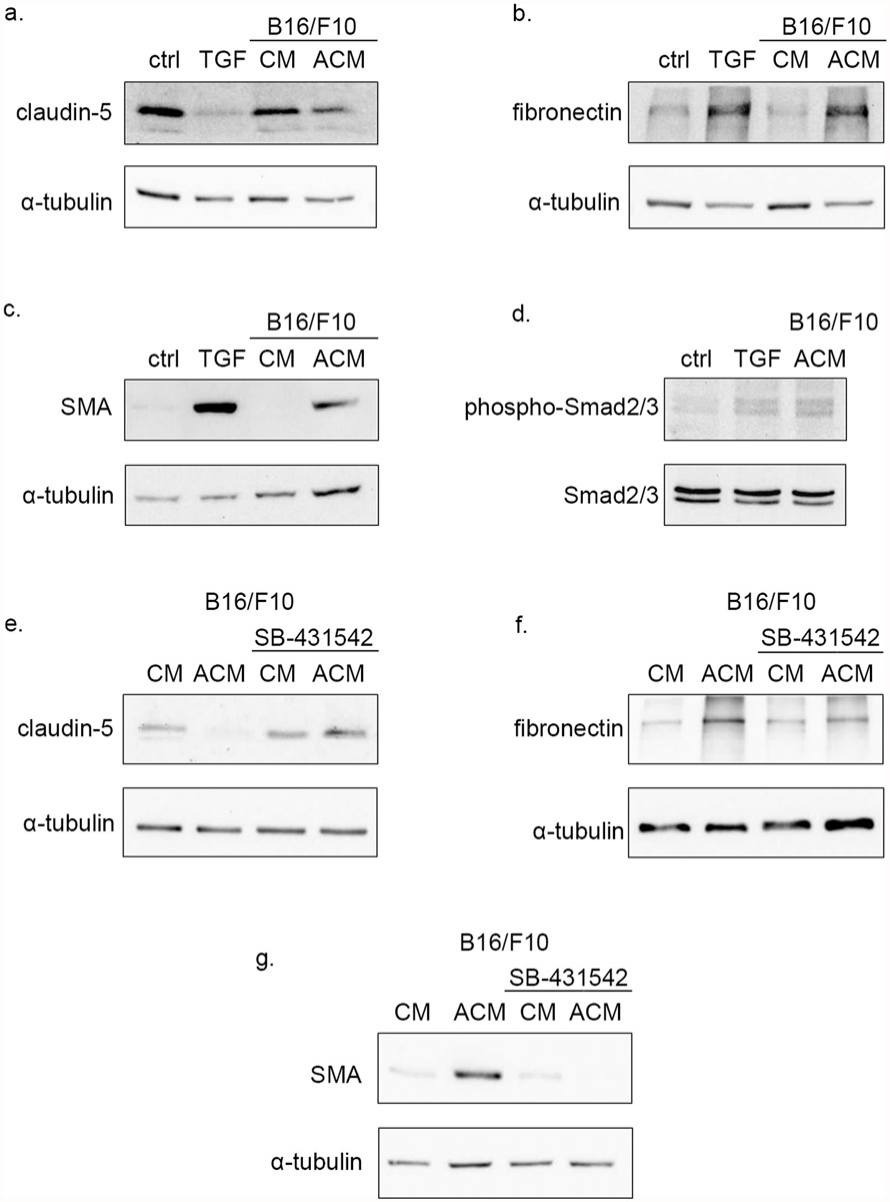
Activated B16/F10 conditioned medium induces EndMT in BECs through TGF-β signaling. PDS-free BEC medium was conditioned with B16/F10 cells for 24 hrs, heat activated and then supplemented with PDS. Conditioned medium (CM) or heat-activated conditioned medium (ACM) was then used to stimulate BECs for 48 hrs, samples being analyzed by Western blot. Similarly to the parallel TGF-β1 treatments, ACM induced EndMT of BECs as evidenced by the reduction of claudin-5 protein levels **(a)**, as well as by the expression of fibronectin **(b)** and SMA **(c)**. **(d)** BECs were stimulated with TGF-β1 or B16/F10 ACM for 30 minutes. Both stimuli induced phosphorylation of Smad2 and Smad3. Cells were pre-treated with 10 μM SB-431542 for 60 min and then treated with conditioned medium (CM) or heat-activated conditioned medium (ACM), in the presence of the inhibitor. Samples were analyzed by Western blot. SB-431542 treatment of RBECs preserved claudin-5 levels **(e)**. The presence of the inhibitor prevented fibronectin **(f)** and SMA **(g)** expression of RBECs when stimulated with B16/F10 conditioned/activated medium.

Pathologic EndMT was described during fibrosis and cancer associated fibroblast differentiation, yet it is less plausible that RBEC EndMT would be relevant for these pathophysiological mechanisms. When we searched for a potential relevance of our findings, we turned our attention towards the interaction between metastatic and endothelial cells. Cancer cells are known to express latent TGF-β [24]. EndMT induced by metastatic cells might be a key step for extravasation through the BBB during metastatic colonization of the brain, but also during extravasation to other target tissues. To assess whether metastatic cells are able to induce RBEC EndMT, RBEC medium was conditioned with B16/F10 murine melanoma cells, TGF-β was then heat-activated and RBECs were stimulated with B16/F10 conditioned medium (CM) or conditioned/activated medium (ACM). Stimulated cells exhibited features of EndMT: similarly to TGF-β1 treatment, B16/F10 ACM, but not CM, down-regulated claudin-5 (Fig. 3a) levels, and induced the expression of fibronectin (Fig. 3b) and SMA (Fig. 3c). Besides TGF-β metastatic and tumor cells are known to express a multitude of growth factors. To show that EndMT induced by cancer cell ACM is mediated by TGF-β signaling, phosphorylation status of Smads was addressed. Similarly to TGF-β1, RBECs responded with phosphorylation of Smad2 and Smad3 when stimulated with B16/F10 ACM for 30 minutes (Fig. 3d). To show that RBEC EndMT induced by metastatic cell ACM is occurring through TGF-β, cells were treated with SB-431542 to interfere with TGF-β receptor signaling. SB-431542 treatment of RBECs preserved claudin-5 levels upon stimulation with B16/F10 ACM (Fig. 3e). The presence of the inhibitor prevented fibronectin and SMA expression of RBECs when stimulated with B16/F10 ACM (Fig. 3f and 3g).

**Fig 4:**
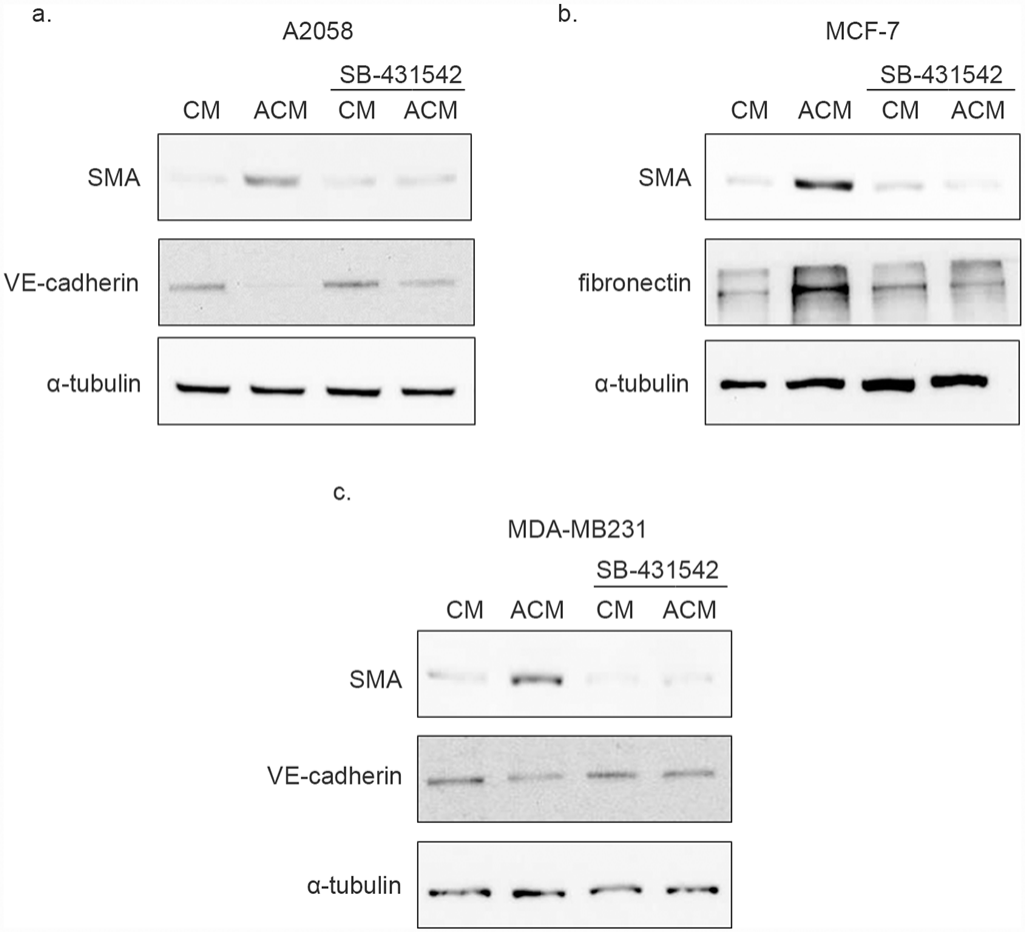
Activated cancer cell conditioned medium induces SMA expression through TGF-β signaling in RBECs. PDS-free BEC medium was conditioned with A2058 human melanoma, MCF-7 human breast cancer or MDA-MB231 human breast cancer cells for 24 hrs. A half of the conditioned media were subjected heat activation, and then both conditioned (CM) and activated conditioned media (ACM) were supplemented with PDS. Cells were pre-treated with 10 μM SB-431542 for 60 min and then treated with CM or ACM, in the presence of SB-431542. Samples were analyzed by Western blot. SB-431542 inhibited A2058 ACM induced SMA expression and VE-cadherin down-regulation **(a)**, as well as MCF-7 ACM induced SMA and fibronectin expression **(b)**. SB-431542 prevented MDA-MB231 ACM induced SMA expression and VE-cadherin down-regulation **(c)**.

Next A2058 human metastatic melanoma cells were used to condition the RBEC medium. RBECs were then treated with A2058 ACM, which induced SMA expression in the cells. SMA expression was prevented in the presence of SB-431542 indicating that A2058 ACM-induced SMA expression was also dependent on TGF-β expressed by A2058 cells. Further, A2058 ACM induced VE-cadherin down-regulation, an effect diminished by the presence of SB-431542 (Fig. 4a).

Finally, to determine whether the effects of metastatic cell ACM are confined to melanoma, or other cancer cell lines could also induce RBEC EndMT, RBEC medium was conditioned with MCF-7 and MDA-MB231 human metastatic breast cancer cells. Following the activation step, MCF-7 ACM induced SMA and fibronectin expression in RBECs, and this effect was mitigated in the presence of the TGF-β inhibitor SB-431542 (Fig. 4b). Similarly, MDA-MB231 ACM induced SMA expression and reduced VE-cadherin levels in RBECs, and these effects could be prevented in the presence of the TGF- β inhibitor SB-431542 (Fig. 4c).

### 3. Metastatic cell conditioned/activated medium reduces transendothelial electric resistance in RBECs in a TGF-β dependent manner

**Fig 5:**
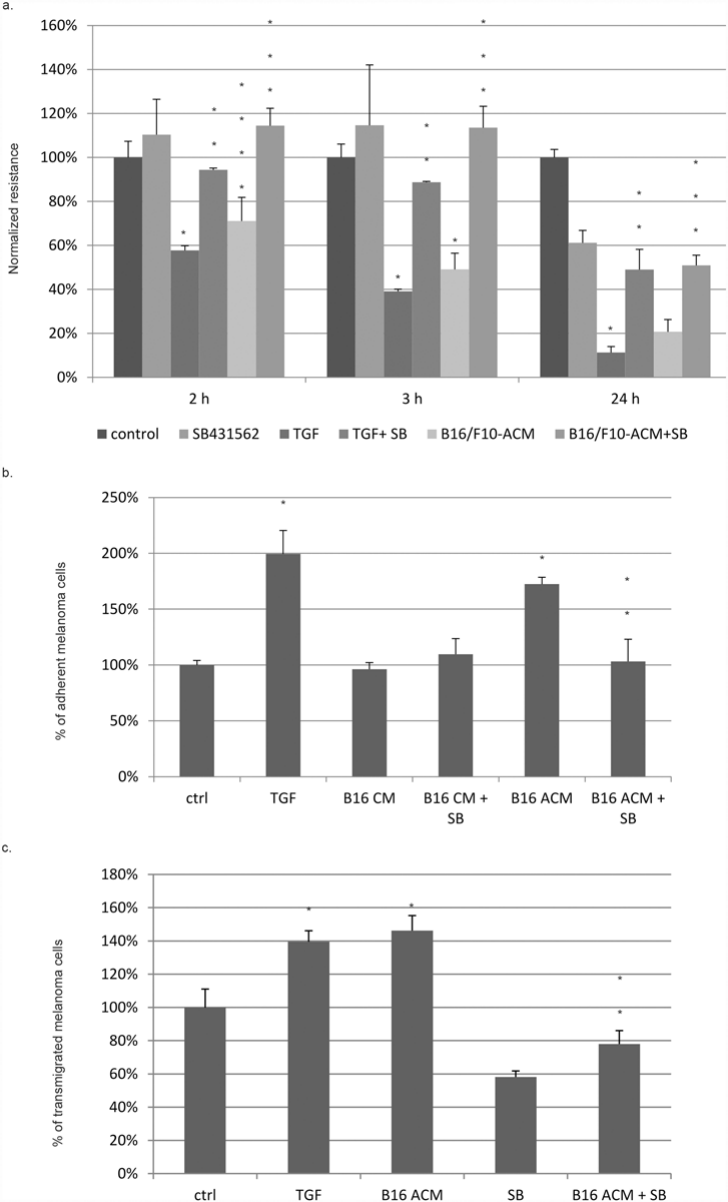
TGF-β1 and activated B16/F10 conditioned medium modulate TEER, melanoma-endothelial cell adhesion and transendothelial migration of melanoma cells. **(a) TGF-β1 and activated B16/F10 conditioned medium reduce TEER of RBECs**. Both TGF-β1 and activated B16/F10 conditioned medium caused a significant, time dependent decline of the transendothelial electric resistance (TEER) of the RBEC monolayer. SB-431542 (60 min) pretreatment and treatment of RBECs inhibited or reduced the decline of TEER upon stimulation with either stimulus. The graph summarizes the results of 3 independent measurements for each treatment, average and SE values are presented. Comparing the TEER between TGF and control (*), ACM and control (*), TGF+SB and TGF (**), ACM+SB and ACM (***) we obtained p<0.05 in all three time points, except for ACM vs. control at 2 hrs where the significance was lower (p<0.01, ****), as assessed by ANOVA and Bonferroni’s post hoc test. **(b) TGF-β-dependent adhesion of B16/F10 melanoma cells to RBECs**. Confluent RBECs were pretreated with TGF-β1, B16/F10 conditioned media (B16 CM) or B16/F10 activated conditioned media (B16 ACM) in the presence or absence of SB-431542 (SB) for 5 hrs. Fluorescently labeled B16/F10 melanoma cells (5x10^4^/well) were plated onto confluent RBECs and left for 70 min. After washing of non-adherent cells, attached melanoma cells were counted. TGF-β1 pretreatment led to a marked increase of melanoma cells attached to the endothelial monolayer. Similarly to TGF-β1, B16/F10 ACM pre-treatment also enhanced the attachment of B16/F10 melanoma cells onto the endothelial monolayer, in a TGF-β- dependent manner, since this effect was mitigated in the presence of the TGF-β inhibitor SB-431542 (p<0.05 in case of TGF vs. control (*), B16 ACM vs. control (*) and B16 ACM+SB vs. B16 ACM (**), as assessed by ANOVA and Bonferroni’s post hoc test). **(c) Enhanced TGF- β-dependent transendothelial migration of melanoma cells**. RBECs were cultured until confluence in 12 well plates and treated with TGF-β1 or B16/F10 ACM for 5 hrs. Fluorescently labelled B16/F10 melanoma cells (2 x 10^4^/well) were plated onto the monolayer. Cells were monitored for 10 hrs and then transmigrating melanoma cells were counted. Both stimuli enhanced the number of transmigrating melanoma cells. Preincubation of RBECs with 10 μM SB-431542 for 60 min inhibited ACM induced transendothelial migration of melanoma cells (*- p<0.01 for TGF vs. control and B16 ACM vs control, **- p<0.01 for B16 ACM+ SB vs. B16 ACM, ANOVA and Bonferroni’s post hoc test).

To test whether TGF-β1 or B16/F10 ACM could induce biomechanical changes and impairment of endothelial junctions that would reduce barrier function and integrity, transendothelial electric resistance (TEER) was measured in RBECs. A marked decrease in TEER was observed already after 2 hrs of TGF-β1 and B16/F10 ACM treatment, and TEER declined further 3 hrs and 24 hrs after treating RBECs. Importantly, pretreatment of RBECs with SB-431542 preserved TEER when stimulated with TGF-β1 or B16/F10 ACM (Fig. 5a). These results showed that TGF-β1 or B16/F10 ACM decreased electric resistance indicating disruption in the RBEC monolayer integrity, and the decline of TEER induced by B16/F10 ACM was TGF-β dependent.

### 4. Metastatic cell conditioned/activated medium enhances adhesion of metastatic cells to endothelial layers

A critical step during metastatic extravasation and transendothelial migration is the adhesion of metastatic cells to the endothelial layer. We wished to assess whether TGF-β could influence the adhesion of metastatic melanoma cells to BECs. The number of attached melanoma cells doubled when endothelial cells were pre-treated with TGF-β1, as compared to the control adhered cell numbers. Further, B16/F10 CM did not affect the number of adhered cells; however, when B16/F10 CM was heat activated, the number of adhered melanoma cells was in the range of the cells adhered upon treatment of BECs with TGF-β1. Importantly, this increase in melanoma-endothelial cell adherence could be reduced in the presence of SB-431542, indicating that the increased adherence between melanoma and endothelial cells is TGF-β dependent (Fig. 5b).

### 5. TGF-β1 and cancer cell ACM enhance transendothelial migration of melanoma cells

To test whether the increase in the number of melanoma cells attached to the endothelium is accompanied by an increase in the number of transmigrating melanoma cells, transmigration assays were performed. Both TGF-β1 treatment and stimulation with B16/F10 ACM significantly enhanced the number of transmigrating B16/F10 melanoma cells (40% and 46% more transmigrating melanoma cells, respectively). Importantly, SB-431542 mitigated this increase in transmigrating cells upon stimulation with B16/F10 ACM, indicating that the ACM induced elevation in transmigrating cell numbers is TGF- β dependent (Fig. 5c).

### 6. Breast cancer cell line conditioned/activated medium induces TGF-β-dependent EndMT in human umbilical vein endothelial cells

**Fig 6:**
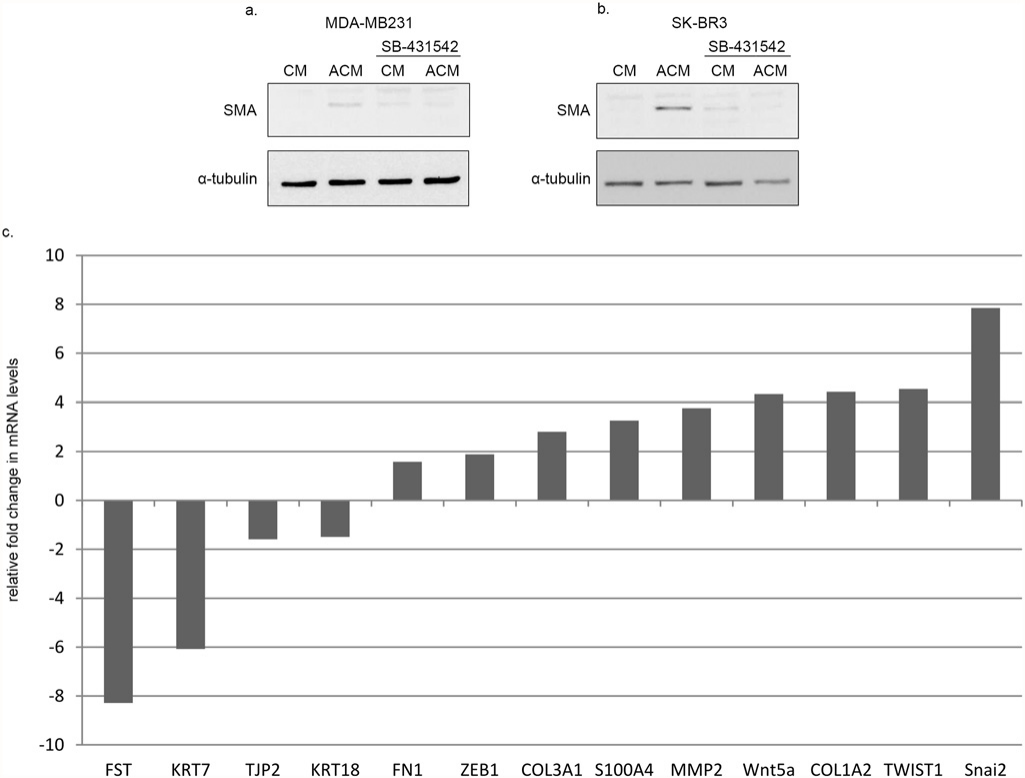
Activated breast cancer cell conditioned medium induces SMA expression through TGF-β signaling in HUVECs. FBS-free M199 medium was conditioned with MDA-MB231 **(a)** or SK-BR3 **(b)** human breast cancer cells for 24 hrs. A half of the conditioned media were subjected heat activation, and then both conditioned (CM) and activated conditioned media (ACM) were supplemented with FBS. Cells were pre-treated with 10 μM SB-431542 for 60 min and then treated with CM or ACM for 72 hrs, in the presence of the inhibitor. Samples were analyzed by Western blot. SB-431542 inhibited MDA-MB231 or SK-BR3 ACM induced SMA expression. **(c) Co-culture with melanoma cells leads to expressional changes characteristic to EndMT in HUVECs**. Gene expression profiles of HUVECs and HUVECs co-cultured with 1205Lu human metastatic melanoma cells were analyzed and compared. In HUVECs co-cultured with melanoma cells there was a marked decrease in expression levels of several endothelial markers (KRT7, KRT18, TJP2), as well as a severe decrease in FST expression. In parallel, co-cultured HUVECs exhibited elevated expression levels of EndMT markers (FN1, COL3A1, S100A4, MMP2, COL1A2) and transcriptional regulators (ZEB1, Wnt5a, TWIST1, Snai2).

Next we sought to investigate whether cancer cells could induce EndMT features in other endothelial cells as well. For this, HUVECs were subjected to stimulation with MDA-MB231 and SK-BR3 human breast cancer cell line conditioned and activated medium. Stimulation of HUVECs with either MDA-MB231 (Fig. 6a) or SK-BR3 (Fig. 6b) ACM, but not CM, resulted in SMA expression. SMA expression was mitigated in both cases in the presence of SB-431542, indicating that these effects were TGF-β dependent.

### 7. Co-culture with melanoma cells leads to expressional changes characteristic to EndMT in HUVECs

To investigate the direct effects of cancer cells on endothelial cells, we performed data mining in publicly available expression profiling databases in the Gene Expression Omnibus (GEO) database. We were searching for an experimental setup relevant to our studies. One such database contained mRNA expression profiles of HUVECs and HUVECs co-cultured with 1205Lu human metastatic melanoma cells. This experimental setup models the direct contact between endothelial and melanoma cells occurring during circulating tumor cell arrest in the microvasculature. We analyzed and compared mRNA expression levels for several additional EndMT markers.

In HUVECs co-cultured with melanoma cells there was a marked decrease in expression levels of several endothelial markers (KRT7, KRT18, TJP2), as well as a severe decrease in FST expression, an EMT/EndMT antagonist. Concomitantly, co-cultured HUVECs exhibited elevated expression levels of EndMT markers (FN1, COL3A1, S100A4, MMP2, COL1A2) and transcriptional regulators (ZEB1, Wnt5a, TWIST1, Snai2) (Fig. 5c).

These results indicate that the presence of melanoma cells in the co-culture induced expressional changes in HUVECs characteristic to an EndMT.

## Discussion

Prior to TEM, the first step of the extravasation process is the attachment of cancer cells to the endothelial cells. The remodeling of endothelial cell contact protein expression is a prerequisite for attachment between cancer and endothelial cells, EndMT ensuring the expression of cognate ligands on endothelial cells which are already expressed by cancer cells. During EndMT BECs lose their intercellular contacts, the expression levels of VE-cadherin, claudin-5 and occludin being diminished or mitigated. The disruption of adherens and tight junctions between neighboring endothelial cells is an important step during TEM since endothelial cell junction opening allows metastatic cells to squeeze between and pass through the endothelial cell layer.

The down-regulation of endothelial cell contact proteins was accompanied by enhanced N-cadherin, β1-integrin and fibronectin expression upon TGF-β1 treatment, this latter gain in protein expression rendering the potential for endothelial cells to attach metastatic cells.

Cadherin switch is an essential step during EMT, when E-cadherin expression is depleted and is followed by de novo expression of N-cadherin [25]. Since endothelial cells express low levels of N-cadherin under control conditions [26], we couldn’t detect a cadherin switch; however in parallel with the decrease of VE-cadherin expression, we observed a marked increase in N-cadherin expression upon EndMT of BECs. N-cadherin is also expressed by cancer cells and it ensures the rolling and attachment of cancer cells to the endothelium [27, 28].

Integrins are a large family of heterodimeric cell adhesion molecules involved in cell-matrix and cell-cell communications. Metastatic cancer cells express integrins, β1-integrin contributing to the adhesion between prostate cancer cells and endothelial cells through its ligand, fibronectin [29]. Moreover, β1-integrin depletion reduced prostate cancer cell adhesion to endothelial cells and fibronectin and reduced extravasation [30]. Fibronectin is a ligand for β1-integrin. Endothelial β1-integrin expression in HUVECs is also required for metastatic cell adhesion [31]. Since cancer cell β1-integrin is important for extravasation, the interaction and attachment of cancer cells to endothelial cells require the presence of β1-integrin and fibronectin on endothelial cells. BECs started expressing β1-integrin and fibronectin upon EndMT suggesting that an EndMT is necessary for efficient cancer cell attachment and extravasation.

Rho GTPases are central regulators of EMT [32, 33, 34], EndMT [35] and of cancer cell adhesion to endothelial cells [30, 36]. Endothelial cells undergo morphological changes and cytoskeletal remodeling during TEM. Endothelial RhoA is activated by lung cancer cell attachment to brain endothelial cells and TEM, which leads to increased actomyosin contractility and actin cytoskeleton reorganization in human brain microvascular endothelial cells [37]. Moreover, inhibition of the Rho/ROCK pathway prevents the disruption of tight junctions in brain endothelial cells [22, 38]. Rho/ROCK is a well-known regulator of SMA expression during EMT and EndMT [35]. BECs stimulated with TGF-β1 responded with calponin and ROCK-dependent SMA expression, rendering enhanced contractile properties corresponding to a myofibroblast-like phenotype, contractility necessary for enabling TEM [39].

When tumor cell conditioned and activated medium was used for stimulation, RBECs expressed fibronectin and SMA, and lost claudin-5 expression in a TGF-β dependent manner, as shown in our experiments. Moreover, both melanoma and breast cancer cell line conditioning had similar effects on RBECs. These effects were not confined to BECs, since breast cancer cell line conditioned and activated medium also induced SMA expression in HUVECs in a TGF-β-dependent manner. TGF-β was responsible for the effects of the ACM, since ACM effects could be inhibited using SB-431542, which exerts its effects on TGF-β1, TGF-β2 and TGF-β3 signaling. Another argument favoring the TGF-β- dependent effects of activated conditioned media from tumor cells is that conditioning alone was not enough to induce EndMT. The heat-activation step was necessary to obtain these effects, and it is well known that TGF-β can be activated by heat or other stimuli. Moreover, ACM stimulation led to phosphorylation of Smad2 and Smad3, a key step during TGF-β1- dependent signaling.

These results were in concordance with our findings in TEER and adhesion experiments. Both stimuli (TGF-β1 treatments, as well as stimulation with tumor cell conditioned/activated medium in a TGF-β dependent manner) induced a reduction in transendothelial resistance, a prerequisite of the disruption of the endothelial layer. Both stimuli enhanced melanoma cell adherence to the endothelial cells, the adherence of the tumor cells to the endothelial cells being probably the first step before TEM. In parallel, and as a consequence of a decrease in TEER and an increase in adhering melanoma cells, both stimuli led to an increase in melanoma cells undergoing transendothelial migration.

TGF-β1 treatments and TGF-β in cancer cell conditioned medium were shown to regulate adhesion between melanoma and endothelial cells, TGF-β1 inducing changes of endothelial morphology [24]. Considering this along with our results on the expressional changes induced TGF-β1 and cancer cell conditioned/activated medium in BECs and HUVECs, the endothelial cell response enabling cancer cell TEM can be termed an endothelial-mesenchymal transition, EndMT being a prerequisite for metastatic extravasation and TEM. Our findings suggest that tumor cell extravasation takes place through a controlled process in which endothelial cells are active facilitators as a consequence of their transition towards a myofibroblastic phenotype. These results indicate that EndMT is one of the potential mechanisms occurring during the complex phenomenon known as metastatic extravasation. During metastasis endothelial cells undergo a rearrangement of cell surface and cell contact proteins, which would correspond to the downregulation of the endothelial program and the activation of the fibroblastic program, whereas the contractility would be conferred by the myogenic program of an EndMT. The transition would not necessarily mean a full conversion into myofibroblastic phenotype, yet the transition would confer the endothelial plasticity required to facilitate metastasis.

TGF-β has a dual role during metastatic progression: it induces the EMT of cancer cells leading to the mobilization of the metastatic population, and it also “preconditions” certain endothelial foci for extravasation through EndMT. Recently it was shown that an EndMT is required for intravasation as well [40], although its extent might be less dramatic since the new blood vessels of the primary tumor vasculature are leaky and generally present weaker endothelial cell-cell junctions [41]. An endothelial to mesenchymal transition-like process was described in lymphendothelial cells during the intravasation of breast carcinoma cells involving the function of EMT inducer ZEB1 [42]. EndMT may be an important step during metastatic extravasation in organs where endothelial organization is more compact. The sinusoid capillaries in the bone marrow [43] and as well as the capillaries of the liver [44] are lined with fenestrated endothelia, which present weaker, more permissive obstacles for the metastatic cells. However, capillaries of other organs (lungs) and the endothelial cells of the BBB [45] are tightly compact, presenting a more restrictive environment for metastasis. Therefore infiltration of metastatic cells into these tissues may require more specialized functions. EndMT induction may be one of such functions along with other mechanisms, such as the release of proteolytic enzymes such as seprase [18], which was found to be regulated by TGF-β as well [46].

There are two possible routes for metastatic cell-induced EndMT. First, metastatic cells, by expressing TGF-β, could exert such an effect locally following an initial contact and a potential weak adhesion step, or during circulating tumor cell arrest in the microvasculature [47]. This latter possibility is being supported by the induction of EndMT during the direct contact between endothelial and melanoma cells in the co-culture experimental setup shown in Fig. 6c. Yet it is important to mention, that in the context of EndMT such an initial weak adhesion could potentially occur mainly through N-cadherin [27, 28], since E-selectin expression is inhibited by TGF-β1 [48]. The other potential mechanism is through a cumulative effect of TGF-β expression and systemic release originating from the primary tumors. This latter possibility is more plausible since some cancer cells were shown to migrate on the endothelium before extravasation, a step that could allow finding the optimal sites for extravasation [49], an observation that could explain how this phenomenon unfolds *in vivo*. Indeed, factors expressed by primary tumors can induce molecular changes in the endothelium before the homing of metastatic cancer cells. Circulating metastatic cells can then later preferentially localize in regions of vascular hyperpermeability [50]. When screening the endothelial wall for the metastatic site, tumor cells screen for the least resistant endothelial point, and EndMT might be the mechanisms leading to this lower resistance.

Current directions for developing therapeutic options to tackle metastatic disease focus mainly on targeting the properties of the malignant cells. The proactive role played by the endothelial cells during intravasation and extravasation could be exploited in the future to reduce metastatic permeability of the endothelial cell barriers.
