# Correction: Endothelial-Mesenchymal Transition of Brain Endothelial Cells: Possible Role during Metastatic Extravasation

**DOI:** 10.1371/journal.pone.0123845

**Published:** 2015-03-30

**Authors:** The PLOS ONE Staff

## Notice of Republication

This article was republished on March 11, 2015, to replace incorrect figures in the PDF that were introduced during the production process. The publisher apologizes for the error. Please download this article again to view the correct version.
